# A Clinical Algorithm for Screening Compensated Advanced Chronic Liver Disease Utilizing Ultrasonography, Platelet Count, and Albumin Levels, With Transient Elastography as Reference

**DOI:** 10.7759/cureus.73879

**Published:** 2024-11-17

**Authors:** Hasaan Rafique, Sadaf Hasaan, Saleha Azhar, Manushri Jain, Majid Khan, Sonika Sethi, Chris Corbett, Saqib Mumtaz

**Affiliations:** 1 Gastroenterology and Hepatology, New Cross Hospital, Wolverhampton, GBR

**Keywords:** cirrhosis, compensated advanced chronic liver disease, diagnosis, hepatology & gastroenterology, screening, transient elastography, ultrasonography

## Abstract

Background and aims

Compensated advanced chronic liver disease (cACLD) refers to asymptomatic patients with advanced fibrosis who do not yet exhibit clinical or radiological signs of portal hypertension. Early detection of cACLD is essential for effective risk stratification and timely management, potentially preventing progression to more severe and irreversible stages of liver disease. Transient elastography (TE) is the primary diagnostic method for cACLD, with several diagnostic thresholds commonly used.

Ultrasonography (USG) is widely used as an initial diagnostic tool for liver disease, but its effectiveness in diagnosing cACLD is not well established. To the best of our knowledge, this study is the first to systematically evaluate the diagnostic accuracy of USG in detecting cACLD, using TE as a reference standard based on validated diagnostic thresholds. We also examined whether combining USG findings with platelet count and serum albumin could enhance diagnostic utility. Additionally, we discuss the strengths and limitations of established non-invasive scoring systems, including the Enhanced Liver Fibrosis (ELF) test, Fibrosis-4 (Fib-4) index, and Nonalcoholic Fatty Liver Disease Fibrosis Score (NFS), to determine if our approach offers a more accessible and practical solution in clinical settings

Methods

This retrospective cross-sectional study was conducted at the Royal Wolverhampton NHS Trust, Wolverhampton, England, including patients with suspected liver disease who underwent USG, TE, and blood tests. Valid TE readings followed manufacturer guidelines, and patients with USG-detected portal hypertension or confounding conditions (e.g., acute hepatitis, heart failure) were excluded. We calculated the sensitivity, specificity, positive predictive value (PPV), and negative predictive value (NPV) of USG against TE, with TE values >15 kPa confirming and <10 kPa excluding cACLD.

Results

Among 1,528 patients (mean age 51 years, range 16-89), 982 were male (64.3%) and 546 were female (35.7%). The cohort predominantly comprised Caucasian (n=1,087, 71.1%) and South-East Asian (n=256, 16.8%) patients. USG showed a sensitivity of 64.6% (95%CI: 58.3-70.6%) and specificity of 78.2% (95%CI: 75.6-80.6%) for cACLD, with a PPV of 40.2% (95%CI: 36.7-43.7%) and an NPV of 90.7% (95%CI: 89.2-92.1%). High NPV was consistent across all etiologies.

In patients with a normal liver on USG, NPV improved to 92.7% (95%CI: 90.9-94.6%) when serum albumin >35 g/L and platelet count >150 x 10^9^/L were present. In patients with sonographic signs of liver disease, PPV increased to 84.1% (95%CI: 73.3-94.9%) when platelet count and albumin were low but dropped to 20.8% (95%CI: 15.4-26.3%) when both were normal.

Conclusions

USG alone has limited reliability in diagnosing cACLD but is valuable for ruling out advanced fibrosis in asymptomatic patients due to its high NPV. Adding platelet and albumin levels improves diagnostic accuracy, though TE remains essential for definitive diagnosis. This approach may streamline screening and optimize resource use, particularly in settings with limited TE access. USG combined with platelet count and serum albumin offers a cost-effective, accessible, and practical solution for the initial assessment of cACLD. Further studies are needed to validate these findings in broader populations.

## Introduction

Cirrhosis represents the end-stage response to chronic liver injury, characterized by the development of thick fibrous bands surrounding regenerative nodules of hepatocytes, leading to substantial architectural distortion of the liver [1]. The Baveno VII consensus describes compensated advanced chronic liver disease (cACLD) as a category that includes asymptomatic patients with significant fibrosis or compensated cirrhosis. These patients do not yet show significant biochemical abnormalities in liver function or radiological indicators of portal hypertension such as ascites, splenomegaly, or varices [2].

Transient elastography (TE), a non-invasive modality using FibroScan® (Echosens, Paris, France), has become a standard tool for assessing liver stiffness in kilopascals (kPa), providing an established and reliable proxy for liver fibrosis severity [3]. Since its validation against liver biopsy in multiple studies [4-6], TE has gained prominence in clinical practice for identifying cACLD. According to the Baveno VII guidelines [2], TE values are interpreted with specific thresholds: a TE value below 10 kPa effectively excludes cACLD, values between 10 and 15 kPa are indeterminate, while a TE above 15 kPa strongly supports cACLD. Higher values, such as those exceeding 25 kPa, are associated with advanced cirrhosis and a higher probability of portal hypertension.

Ultrasonography (USG) is widely accessible and commonly used as a first-line imaging modality in the evaluation of abnormal liver function and suspected chronic liver disease [7]. However, studies assessing the accuracy of USG for diagnosing cirrhosis have shown variable sensitivity and specificity [8-14], with significant inter-observer variability. While early detection of cirrhosis in asymptomatic patients has potential prognostic benefits, false-positive results from USG may lead to unnecessary, often invasive follow-up procedures like liver biopsy or endoscopy. Despite these limitations, USG remains integral to routine liver disease evaluation due to its accessibility and cost-effectiveness. However, no peer-reviewed studies to date have directly evaluated the diagnostic performance of USG compared to TE specifically for detecting cACLD, especially in a wide range of aetiologies.

The aim of this study is to assess the diagnostic accuracy of USG in identifying cACLD, using TE values as the reference standard based on Baveno VII-defined cut-offs. We also investigate whether combining USG findings with easily accessible blood markers, such as platelet count and serum albumin, improves diagnostic utility, potentially providing an efficient, cost-effective, and accessible screening approach. Additionally, we discuss our approach relative to other non-invasive scoring systems, including the Enhanced Liver Fibrosis (ELF) test, Fibrosis-4 (Fib-4) index, and Nonalcoholic Fatty Liver Disease (NAFLD) Fibrosis Score (NFS), to determine if it offers a more practical solution for initial assessment of liver fibrosis.

## Materials and methods

Study design

This retrospective cross-sectional study was conducted at the Department of Gastroenterology, New Cross Hospital, Royal Wolverhampton NHS Trust, Wolverhampton, United Kingdom (UK). The study included all the patients with suspected chronic liver disease who had undergone abdominal USG, TE, and blood tests at any time between December 2013 and March 2020. This study was approved by the Royal Wolverhampton NHS Trust’s Research and Development Department. As a retrospective case review, formal ethical approval was deemed unnecessary. The project was classed as Service Evaluation and as such a written agreement was obtained from the Consultant, Department of Enterology, The Royal Wolverhampton NHS Trust, dated April 13, 2020.

Patient selection

TE has been in clinical use at our institution since 2013, with assessments conducted by a specialist nurse certified by the manufacturer (Echosens). We included all patients with valid TE readings, based on the following manufacturer’s criteria: (1) at least 10 valid shots, (2) a success rate (ratio of valid to total shots) above 60%, and (3) an interquartile range (IQR) of less than 30% of the median liver stiffness measurement (LSM). Patients were eligible if they had both TE and USG completed within a 12-month interval. When multiple USG or blood test results were available within that period, the ones nearest to the TE date were selected for consistency.

Patients were excluded if the USG report indicated signs of portal hypertension such as ascites, splenomegaly, varices, hepatofugal flow in the portal vein, or recanalization of the umbilical vein, as well as any evidence of biliary disease, or if their records revealed confounders likely to interfere with valid TE readings such as acute hepatitis (ALT >250 IU/L), severe heart failure (ejection fraction <30% on recent echocardiography), or prior episodes of hepatic decompensation. Patients with a liver transplant were also excluded.

USG criteria

For this study, USG reports were categorised based on specific terminology agreed with sonographers. Features indicative of possible cirrhosis included coarse echo texture, irregular liver margins, and nodular or heterogeneous parenchyma. USG reports that did not mention these findings were categorised as "not suggestive of cirrhosis."

Data collection

Demographic data, liver disease aetiology, TE and USG findings, and results of blood tests (platelet count and serum albumin levels) were extracted from hospital records. A comprehensive spreadsheet was used for data entry and quality control.

Statistical analysis

We used TE as the reference standard for diagnosing compensated advanced chronic liver disease (cACLD), applying Baveno VII cut-offs where a TE value >15 kPa confirmed and <10 kPa excluded cACLD. For each diagnostic method, we calculated sensitivity, specificity, positive predictive value (PPV), and negative predictive value (NPV). Additional analyses assessed whether the inclusion of platelet count (<150 x 10^9^/L) and serum albumin (<35 g/L) in conjunction with USG findings influenced predictive accuracy. Statistical analyses were performed using MedCalc® Statistical Software version 19.6 (MedCalc Software Ltd, Ostend, Belgium), with p-values <0.05 considered statistically significant.

## Results

Study cohort characteristics

Out of 3,357 TE procedures conducted at the Royal Wolverhampton NHS Trust between December 2013 and March 2020, 2,597 met the validity criteria. From these, 1,973 patients had a corresponding USG examination within 12 months of their TE. Following exclusions (145 patients with USG-detected portal hypertension, 246 with more than one TE in a 12-month period, and 54 with confounding factors like decompensation, acute hepatitis, or heart failure), the final analysis included 1,528 patients.

The mean age of participants was 51 years (range: 16-89), with 982 males (64.27%) and 546 females (35.73%). The most common ethnicities were Caucasian patients (n=1087, 71.14%), followed by South-East Asian patients (n=256, 16.75%). Common liver disease aetiologies were alcohol-related liver disease (ALD) (n=312, 20.4%) and NAFLD (n=452, 29.5%). Other conditions included hepatitis C (n=175, 11.5%), hepatitis B (n=168, 11%), primary biliary cholangitis (n=63, 4.1%), autoimmune hepatitis (n=42, 2.7%), and haemochromatosis (n=41, 2.7%), with the remainder classified as non-specific (n=275, 18.00%).

Diagnostic accuracy of USG for cACLD

USG demonstrated a sensitivity of 64.63% (95%CI: 58.3-70.6%) and a specificity of 78.18% (95%CI: 75.6-80.6%) in detecting cACLD, with a PPV of 40.15% (95%CI: 36.71-43.70%) and a high NPV of 90.71% (95%CI: 89.15-92.06%). These results underscore USG’s limited reliability in confirming cACLD and its usefulness as a tool for ruling out advanced fibrosis.

Subgroup analysis across aetiologies

The predictive values of USG were largely consistent across various liver disease aetiologies. Table 1 shows the sensitivity, specificity, PPV, and NPV of USG for diagnosing cACLD across a range of aetiologies.

**Table 1:** The sensitivity, specificity, and predictive values of u;trasonography in detecting compensated advanced chronic liver disease compared across various aetiologies. ALD: alcoholic liver disease; NAFLD: non-alcoholic fatty liver disease; AIH: autoimmune hepatitis; PBC: primary biliary cirrhosis; HH: hereditary haemochromatosis

Aetiology		Sensitivity	Specificity	Positive predictive value	Negative predictive value
All (n=1528)	%	64.63%	78.18%	40.15%	90.71%
	95% CI	58.31% to 70.60%	75.60% to 80.60%	36.71% to 43.70%	89.15% to 92.06%
	p	<0.0001	<0.0001	<0.0001	<0.0001
ALD (n=312)	%	77.17%	57.86%	51.45%	81.42%
	95% CI	67.25% to 85.28%	49.79% to 65.64%	46.12% to 56.74%	74.63% to 86.71%
	p	<0.0001	<0.0001	<0.0001	<0.0001
NAFLD (n=452)	%	55.95%	86.08%	52.22%	87.79%
	95% CI	44.70% to 66.78%	81.72% to 89.74%	43.85% to 60.47%	84.91% to 90.18%
	p	<0.0001	<0.0001	<0.0001	<0.0001
Hepatitis B (n=168)	%	100%	85.62%	8.00%	100%
	95% CI	15.81% to 100.00%	79.22% to 90.66%	5.62% to 11.26%	-
	p	1	<0.0001	<0.0001	-
Hepatitis C (n=175)	%	48.65%	83.33%	48.65%	83.33%
	95% CI	31.92% to 65.60%	75.20% to 89.66%	35.86% to 61.62%	78.33% to 87.37%
	p	0.0001	<0.0001	<0.0001	<0.0001
AIH (n=42)	%	75%	73.08%	30%	95%
	95% CI	19.41% to 99.37%	52.21% to 88.43%	15.49% to 50.05%	77.40% to 99.06%
	p	0.49	0.019	<0.0001	0.42
PBC (n=63)	%	50%	62.50%	5.26%	96.77%
	95% CI	1.26% to 98.74%	47.35% to 76.05%	1.31% to 18.89%	88.06% to 99.19%
	p	0.54	0.0001	<0.0001	0.28
HH (n=41)	%	66.67%	83.87%	44.44%	92.86%
	95% CI	22.28% to 95.67%	66.27% to 94.55%	23.05% to 68.11%	80.58% to 97.60%
	p	0.27	0.05	0.003	0.13
Non-specified (n=275)	%	70%	76.99%	20.29%	96.84%
	95% CI	45.72% to 88.11%	71.12% to 82.17%	14.97% to 26.91%	93.99% to 98.36%
	p	0.03	<0.0001	<0.0001	0.005

Impact of platelet count and albumin on predictive accuracy

When platelet count (<150 x 10^9^/L) and serum albumin (<35 g/L) were incorporated into USG findings, the PPV for detecting cACLD increased significantly to 81.3% (95%CI: 71.7-90.8%, p <0.0006). In contrast, patients with normal platelet and albumin levels showed a reduced PPV of 21% (95%CI: 15.2-26.6%, p <0.0001). The NPV of USG for excluding cACLD was high at 92.7% (95%CI: 90.9-94.8%, p <0.0001) when both platelet count and albumin were within normal ranges (Table 2). Patients in this group exhibited a median liver stiffness measurement (LSM) of 6.0 kPa (IQR 3.3) in our study. These findings suggest that combining these blood markers with USG enhances diagnostic accuracy, making USG a more reliable tool for cACLD detection.

**Table 2:** The effect of platelet count and serum albumin level on predictive values of USG for diagnosing compensated advanced chronic liver disease. PPV: positive predictive value, NPV: negative predictive value

Lab parameters	Cirrhosis suggested on USG			No cirrhosis on USG		
	n	PPV		n	NPV	
Platelet count <150 x 10^9^/L	134	%	68.64%	72	%	71.87%
		95% CI	60.27% to 77.01%		95% CI	60.86% to 82.89%
		p	<0.0001		p	<0.0001
Platelet count <150 x 10^9^/L and Albumin <35 g/L	48	%	84.09%	14	%	54.55%
		95% CI	73.28% to 94.90%		95% CI	25.12% to 83.97%
		p	0.0086		p	0.048
Platelet count >150 x 10^9^/L	347	%	28.06%	918	%	92.10%
		95% CI	22.78% to 33.34%		95% CI	90.26% to 93.94%
		p	<0.0001		p	<0.0001
Platelet count >150 x 10^9^/L and Albumin ≥35 g/L	271	%	20.83%	855	%	92.74%
		95% CI	15.42% to 26.25%		95% CI	90.91% to 94.57%
		p	<0.0001		p	<0.0001

Differential PPV of various sonographic findings

Coarse echotexture, either on its own or in combination, was the most frequently reported positive finding on USG (n=359, 43.7%). In our analysis, it showed an overall PPV of 37.67% (95%CI: 32.10-43.15%, p <0.0001) for detecting cACLD. This reduced to 22% (95%CI: 14.10-29.53%, p<0.0001) when it was the only positive finding; and dropped further to 13.23% (95%CI: 5.18-21.29%, p<0.0001) when associated with normal platelet count and serum albumin. This cohort in our study had a median LSM of 7.5 Kpa (IQR 5.4). Irregular liver margin was the least frequent positive finding on USG (n=44, 5.3%); however, when present, it had the highest PPV at 92.50% (95%CI 84.33% to 100%, p=0.0083).

## Discussion

Abdominal USG is a valuable but imperfect tool for the evaluation of chronic liver disease. It is known to be very effective in identifying signs of portal hypertension and decompensation, which can often be the first presentation of patients with liver disease; however, its role in the diagnosis of cirrhosis in patients who are asymptomatic is not well established [8-17].

Our study positions USG as a valuable initial tool in identifying patients with cACLD, particularly as a cost-effective, accessible option in primary care and resource-limited settings. While USG alone demonstrated limited sensitivity in confirming cACLD, its high NPV of over 90% suggests that it is a reliable tool for excluding advanced fibrosis in asymptomatic patients.

We found that the addition of platelet count and serum albumin concentration to the findings of USG had a significant effect on the predictive values. This is not unsurprising as a low platelet count is the most common haematological abnormality found in patients with chronic liver disease and is an indicator of advanced disease [18,19]. Similarly, albumin is a protein that is produced only in the liver and is considered a marker of the synthetic function of the liver. The rationale for incorporating these two serum markers is their common availability as screening blood tests and their use in validated risk stratification scores [20-22].

Comparative analysis with TE

TE is a well-validated non-invasive measure for assessing liver fibrosis. TE values above 15 kPa strongly indicate cACLD and values above 25 kPa are highly suggestive of portal hypertension, as outlined in the Baveno VII consensus [2]. Although liver biopsy remains the gold standard method to diagnose cirrhosis, its susceptibility to sampling error and risk of complications limits its use in asymptomatic patients [23-25]. Our study used TE as the reference standard and found that USG alone does not match TE’s diagnostic accuracy; however, USG remains valuable as an initial screening tool, especially where TE access is limited. By combining USG with blood markers such as platelet count and serum albumin, we improved the PPV of USG for cACLD from 40% to 81.3% (p <0.0006). This suggests that, while USG may not replace TE, it can be a useful triage tool to identify patients who may benefit from further assessment with TE.

Comparison of our approach with other non-invasive markers

ELF Score

The ELF score combines serum markers, including hyaluronic acid, procollagen III amino-terminal peptide, and tissue inhibitor of metalloproteinases-1, to assess liver fibrosis. This test has demonstrated high sensitivity for detecting advanced fibrosis; however, it requires specialized assays, making it costly and limiting its accessibility outside of tertiary centres. Additionally, the ELF score is primarily validated for use in NAFLD and chronic hepatitis C populations, which restricts its general applicability across diverse liver disease aetiologies [20]. *Fib-4 Index*

The Fib-4 index, which uses age, alanine transaminase (ALT), aspartate aminotransferase (AST), and platelet count, is widely used for fibrosis assessment, particularly in hepatitis C and NAFLD. It has shown reasonable diagnostic accuracy for detecting significant fibrosis and is especially helpful for ruling out advanced fibrosis in low-risk patients. However, the Fib-4 index is less specific in patients over 65 years of age, and its reliability decreases in asymptomatic patients or those with early-stage liver disease, as often seen in cACLD [21]. Our study’s approach with USG combined with platelet and albumin levels provides a targeted, practical alternative that may be more reliable in asymptomatic populations. Further studies could be done to compare this directly.

NFS

The NFS includes variables such as age, body mass index (BMI), hyperglycemia, platelet count, albumin, and AST/ALT ratio, and is specifically validated for NAFLD. It is a valuable tool for assessing fibrosis in NAFLD populations, though its accuracy may vary due to metabolic factors, limiting its utility in mixed-etiology cohorts. In contrast, our approach, combining USG with platelet count and serum albumin levels, demonstrated reliable predictive values across various etiologies, supporting its broader applicability [22]. More studies may be needed in this area.

Implications for clinical practice

**Figure 1:**
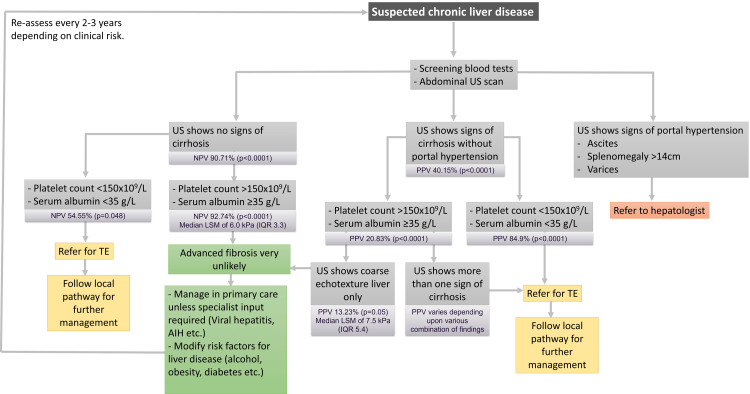
Suggested approach to the evaluation of a suspected case of liver disease using ultrasonography, serum albumin, and platelet count TE: transient elastography; LSM: liver stiffness measurement; IQR: interquartile range; PPV: positive predictive value; NPV: negative predictive value; US: ultrasonography; AIH: autoimmune hepatitis Image Credit: Hasaan Rafique (author)

Our findings support the use of USG, in combination with platelet and albumin levels, as a practical initial screening method for cACLD. This approach is particularly beneficial in primary care or low-resource settings where TE and advanced blood assays may be unavailable. By using USG and basic blood tests to rule out cACLD in low-risk patients, healthcare providers can reserve TE for those at higher risk, potentially reducing costs and improving the efficiency of liver disease screening programs. This tiered diagnostic algorithm aligns with a resource-conscious approach, allowing clinicians to identify patients who may benefit from further evaluation without overburdening healthcare systems with unnecessary testing (Figure 1).

Limitations

The retrospective design and reliance on TE, rather than liver biopsy, as the reference standard are notable limitations of this study. While TE is well-validated for assessing liver fibrosis, biopsy remains the definitive diagnostic tool, albeit less feasible in routine practice due to its invasive nature and associated risks. Additionally, USG is operator-dependent, and inter-observer variability may influence its diagnostic performance. Although standardized terminology was used to categorize USG findings, this variability remains a limitation. Future studies with a prospective, multicenter design and biopsy confirmation could further validate and refine this diagnostic algorithm.

Future directions

Prospective studies are warranted to validate these findings across broader patient populations and in primary care settings. Additionally, investigating whether further blood markers, such as AST/ALT ratios or other routine biochemical parameters, could enhance the predictive accuracy of USG would be valuable. Exploring machine learning or artificial intelligence-driven algorithms to analyze USG findings in conjunction with clinical and biochemical data could also offer a promising avenue for improving non-invasive liver disease assessment.

## Conclusions

This study demonstrates that while USG alone is not sufficiently reliable to diagnose cACLD, it serves as an effective exclusionary tool due to its high NPV. Incorporating platelet count and albumin values into initial assessments enhances USG’s diagnostic accuracy, creating a practical approach for primary screening. It provides an efficient pathway to identify patients who may benefit from further non-invasive assessment with TE, supporting a more streamlined, resource-conscious approach to cACLD diagnosis.

Our findings suggest a potential diagnostic algorithm for cACLD screening in clinical practice. Implementing USG as a first-line tool with supplementary blood markers may reduce unnecessary TE use, optimising healthcare resources. Further prospective studies are encouraged to validate these results and refine non-invasive diagnostic strategies for liver disease.
